# DBSCAN-Based Tracklet Association Annealer for Advanced Multi-Object Tracking

**DOI:** 10.3390/s21175715

**Published:** 2021-08-25

**Authors:** Jongwon Kim, Jeongho Cho

**Affiliations:** Department of Electrical Engineering, Soonchunhyang University, Asan 31538, Korea; jw.kim@sch.ac.kr

**Keywords:** object tracking, DBSCAN, video surveillance, trajectory separation, clustering

## Abstract

Recently, as the demand for technological advancement in the field of autonomous driving and smart video surveillance is gradually increasing, considerable progress in multi-object tracking using deep neural networks has been achieved, and its application field is also expanding. However, various problems have not been fully addressed owing to the inherent limitations in video cameras, such as the tracking of objects in an occluded environment. Therefore, in this study, we propose a density-based object tracking technique redesigned based on DBSCAN, which has high robustness against noise and is excellent for nonlinear clustering. Moreover, it improves the noise vulnerability inherent to multi-object tracking, reduces the difficulty of trajectory separation, and facilitates real-time processing through simple structural expansion. Through performance test evaluation, it was confirmed that by using the proposed technique, several performance indices were improved compared to the existing tracking technique. In particular, when added as a post processor to the existing tracker, the tracking performance owing to noise suppression was considerably improved by more than 10%. Thus, the proposed method can be applied in industrial environments, such as real pedestrian analysis and surveillance security systems.

## 1. Introduction

Recent advances in autonomous driving, intelligent robots, and smart video surveillance systems have evidenced multi-object tracking (MOT), which aims to estimate the trajectories of multiple objects of interest identified over time in a video sequence, as one of the most important computer vision tasks [1,2]. The predominant approach developed for MOT over the past few years is a new paradigm that identifies objects based on deep neural networks (DNNs) and draws trajectories by analyzing target identities in videos. Most MOT algorithms, designed based on a tracking-by-detection method, have achieved significant performance improvement over conventional methods using feature descriptors, such as the scale-invariant feature transform [3] or histogram of oriented gradients [4].

A tracking-by-detection framework is mainly divided into two phases: the object detection step, which locates the target in the current frame of a video, and the association of all detections throughout the frames and existing trajectories. Because DNNs can secure high object detection performance [5,6], most of the recently proposed MOTs focus on solving the data association problem to describe the path of each object over time by assigning an object identification number and clustering tracklets. Tracklets are a small set of paths associated with individual detections in consecutive frames [7,8]. Various techniques, such as prediction filters, graph-based optimizers, and shortest path methods, are generally combined to improve the efficiency of the data association process. In this process, the similarity of the predicted objects is analyzed, a trajectory is created, and an identity number is assigned when the objects detected in succession are the same. Recently, DNNs have been used as embedding vector extractors for object similarity determination [9,10].

**Figure 1:**
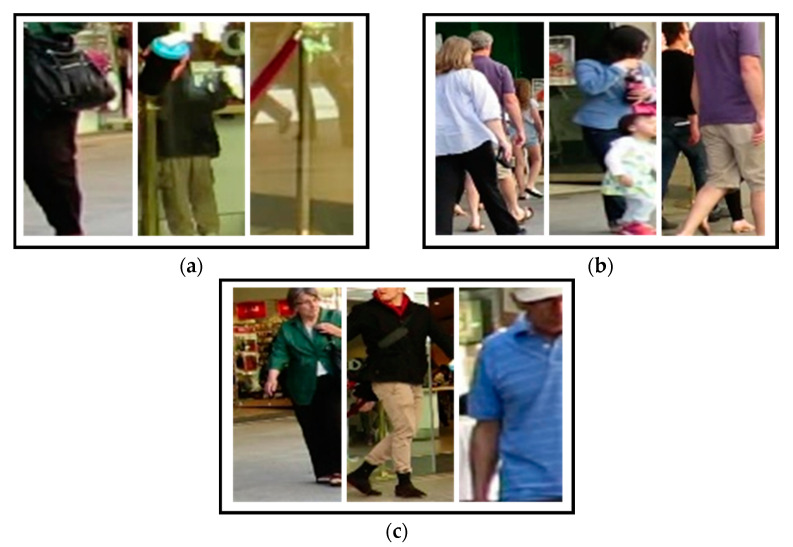
Examples of cases that make object detection difficult when tracking multiple objects, causing problems with accuracy: (**a**) false detection, (**b**) temporal occlusion, (**c**) noisy detection.

Despite such significant technological advances, some challenges still need to be resolved. The first typical problem is false detection, which results in incorrectly drawing the trajectory of a detected object. This may be caused by the low reliability of the detector, where detected objects are regarded as noise in the association process, resulting in lower tracking performance and increased unnecessary computation. The use of DNNs has resulted in a decrease in the false detection rate, but false detection is still an important issue arising from the influence of the surrounding environment or noise. The second problem is the temporal occlusion caused by obstacles or joint paths, which is a consequence of the limited field of view (FoV) of a video camera. As a result, the trajectory of the object is separated owing to the loss of detections, which is called fragmentation (Frag), and two trajectories are created in one ground truth because of the transfer of the ID by an adjacent object, which is called ID switch (IDS). Finally, there is the noisy detection problem. The MOT estimates the trajectory of the target by connecting the bounding box of the object detected in the current frame with that of the object detected in the previous frame. In this process, owing to view variations due to weather, spatial misalignment, changes in the size and position of various objects, etc., the detector unintentionally predicts the bounding box, including noise. Figure 1 shows examples of the MOT related problems described above.

Because the aforementioned problems are inevitable, creating an ideally compact bounding box to perfectly estimate when the detector finds the object and that is not affected by the FoV is not possible. Recent emerging state-of-the-art techniques concentrate on raising the trajectory completeness to the limit where the problems caused by Frag or IDS [11] can be offset. Nevertheless, these obstacles can still cause fatal statistical errors in practical fields that require specialized statistical analysis indicators, such as systems that analyze pedestrian behaviors or track specific objects. This should be a future direction for improving the MOT technique. In this regard, we designed a scheme similar to the existing graph model to solve the Frag and IDS problems in practical applications. 

The graph-based tracking algorithm has been implemented by associating object trajectories between frames using graphs [12,13] or associating clustered trajectories for a short period of time [14]. The object in the graph-based model is defined as a node, and the similarity between objects is defined as the edge weight. Therefore, the similarity between the interconnected nodes was determined based on their edge weights. The optimization of the edge weights has been conducted to minimize the total cost by applying the distance between objects [15,16], joints [17], and motions [18]. The graph-based tracking algorithm has been applied to various weighted graph models and has shown superiority in short tracklet connections. However, a limitation arises when trying to connect a path over a wide area, and the problem of overlapping objects remains because the pertinent feature of the object expressed in a real situation is not considered. In addition, the computational amount of the affinity matrix becomes excessive during the process of extracting and optimizing the edge weight, hindering real-time implementation [19].

The MOT technique based on the features of the detected object was proposed after the development of the DNN, and it was followed by feature vector embedding [20,21]. Although various similarity models have been proposed, such as modifying the convolutional neural network (CNN) structure [22,23] or vector embedding through fusion with long short-term memory [24,25], many studies still use feature vectors and graph models extracted through reidentification-based CNN [26,27]. They have higher robustness than graph models in an environment where temporal occlusion or noise exists, but vast amounts of data must be secured for learning. In addition, these models do not address the problem of changing IDs between objects when there is considerable overlapping of objects in a crowded environment within the frame or when objects have similar colors.

An IOU Tracker [28] was proposed for object tracking by dividing objects between frames using the intersection-over-union (IOU) threshold of the bounding box. Although its structure is simple, it highly depends on the detection result, and if there is a temporal occlusion, such as when the objects are too close to each other or are partially occluded by other objects, the reliability rapidly decreases. The simple online and real-time tracking (SORT) approach [29] was also proposed and used to estimate the velocity of an object and predict its position in the next frame. After prediction through the Kalman filter, the predicted and detected values are associated with the Hungarian algorithm [30] to complete the trajectory of the object. This approach is similar to the IOU tracker, but it solves the temporal occlusion problem by predicting the object location. Later, similar model-based methods using feature vectors, such as object motion, joints, and optical flows, were proposed and advanced [31,32,33]. However, limitations in the predictive model make it vulnerable to noisy environments and false detections, and the structural complexity is increased.

**Figure 2:**
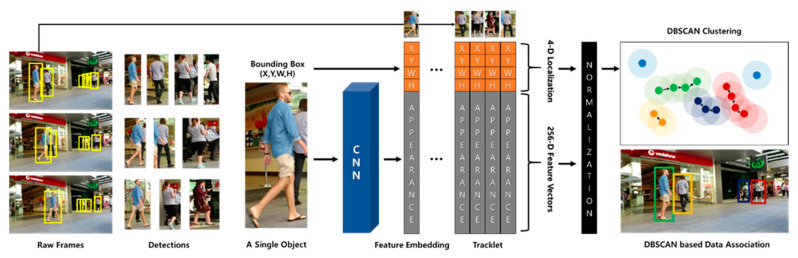
Workflow of the proposed MOT scheme.

In this study, we propose a density-based tracklet association annealer (DTAA)—a novel tracking-by-detection technique. This technique aims to improve the vulnerability to noise and difficulty of trajectory separation inherent to MOT and to secure real-time processing through simple structural expansion. In addition, it can effectively reduce IDS and Frag in tracking. The proposed DTAA extends the clustering mechanism of density-based spatial clustering of applications with noise (DBSCAN) [34], which has high noise robustness and is excellent for nonlinear clustering, to a graph model. Although DBSCAN is a spatial data clustering technique, we have proven in prior research [35] that an adaptive graph clustering technique based on DBSCAN shows high performance in clustering real-world data. In addition, we confirmed that the spatial data clustering technique could be analyzed in the coordinate system of two-dimensional (2D) images in a previous study [36]. Based on the results of these preliminary studies, the proposed DTAA combines the coordinates of the bounding box with the feature vectors of the detected objects and extracts them as embedding vectors for clustering. The reduced-dimensional feature vectors are clustered into highly similar tracklets via DBSCAN, and heterogeneous tracklets or single objects are eliminated. The workflow of the proposed tracking scheme is shown in Figure 2.

The proposed DTAA has advantages comparable to or more than conventional density-based clustering techniques. First, it is robust against false positives. DTAA can filter false detections from clusters in real time. Second, with a relatively simple structure, it can be used independently or as a postprocessor to rebuild the existing MOT techniques, in which the Frag and IDS indicators can be significantly reduced. Finally, because the feature vectors are created through the fusion of the location and similarity of the identified object, spatial features and similarities are used for end-to-end clustering. To evaluate the robustness of the proposed tracking strategy, the MOT15-16 [37,38] benchmarks were used with the addition of various artificial noises, and a comparative evaluation was performed with the latest techniques using DNN. As a result, when the proposed DTAA was added to the existing tracking technique as a postprocessor, the performance of the existing tracking system was improved by more than 5% on average, and the Frag and IDS indices were decreased by more than 30–40%. It was further confirmed by testing in the presence of additional noise that the robustness against noise was improved compared to the existing tracking technique. In addition, in all experiments conducted in NVIDIA GTX 1080Ti and Intel Core i7-8700 CPU environments, it showed a near real-time processing speed of 20 frames per second (FPS) or more, and it is expected that the real-time anomaly detection system will be used in practical applications in the future.

The contributions of this study are summarized as follows:

1. A new density-based tracklet association enhancement method for improving the performance of “multi-object tracking by detection” is proposed.
2. A clustering technique suitable for multi-object tracking is introduced that combines the reduced-dimensional feature vectors obtained through CNN into very similar tracklets using DBSCAN.
3. When tracking multiple objects, the severe vulnerability to small external interference and the difficulty of separating multi-object trajectories were improved, and real-time multi-object tracking became possible through simple structure extension.

## 2. Density-Based Tracklet Association

The proposed DTAA extracts object features through conventional person re-identification (re-ID) utilizing a CNN. It is composed of the edges of the track graph by embedding the extracted features and object bounding box coordinates. In the process of data association, DBSCAN is applied instead of a graph model to solve the problem of computational complexity of the similarity matrix through edge clustering rather than graph model optimization. As a result, it is feasible to collect tracks for a longer period of time than that of the existing computational amount, and tracks that are considered unnecessary noise in real-time clustering are deleted. Therefore, the existing optimization problem can be approached more reasonably and efficiently.

### 2.1. DBSCAN

DBSCAN is a method of clustering data points that share common attributes based on the density of data, unlike most techniques that incorporate similar entities based on their data distribution. This means that clusters are defined as events occurring in the same space. This method is suitable for the clustering of multidimensional and spatial data based on the density correlation with neighboring clusters, and data containing various sizes, shapes, or noises can also be clustered [39]. Unlike the most commonly utilized k-means clustering, DBSCAN does not require the number of clusters in advance, and it receives only two hyperparameters. One is the minimum neighboring radius, *ϵ*, which means the area in density and is defined as the distance from which data is viewed as a neighbor. Assuming that there are two data points *x* and *x*’, and the distance between them is *d*, if the condition *d*(*x*, *x*’) ≤ *ϵ* is satisfied, *x* and *x*’ are included in the same cluster. The other is *minPts*, which is the minimum number of points in the area defined above. A cluster is then formed when more than the minimum number of points is satisfied within a specific area based on the minimum neighboring radius.

**Figure 3:**
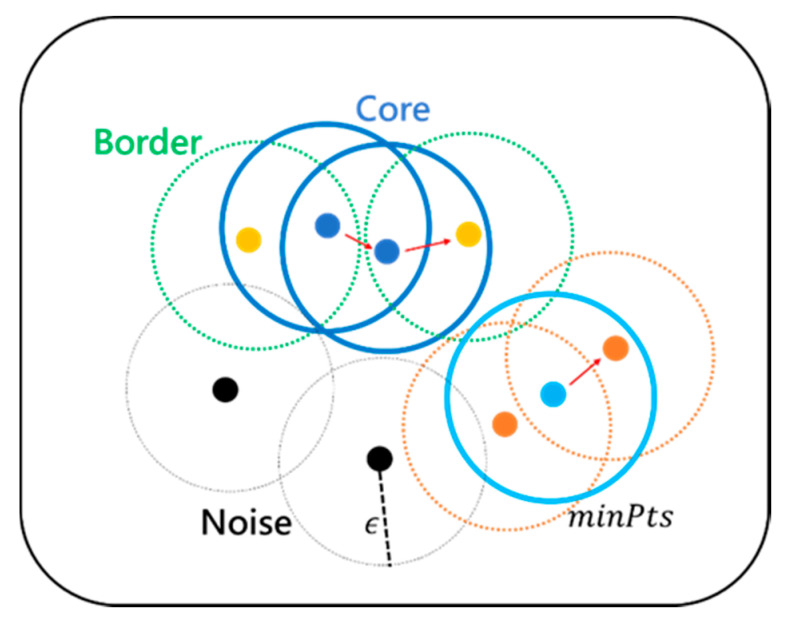
Clustering element vectors of DBSCAN.

The cluster is expanded by repeating this process around a vector neighboring the newly formed cluster. Here, a central vector that contributes to cluster formation is defined as a core vector, which refers to a case in which the number of neighboring vectors within the radius *ϵ* from an arbitrary vector is higher than *minPts*. A vector that is a neighbor of the core vector but cannot itself become a central vector is defined as a border vector. It is located within the distance *ϵ* from the core vector and is classified into the same cluster, but it is a vector located outside the cluster. A vector that is not a core or outer vector, that is, a vector whose number of neighboring vectors is lower than the number of *minPts* within *ϵ* and does not belong to any cluster, is considered to be a noise vector. A description of these vectors is presented in Figure 3. Because DBSCAN clusters use the density of neighboring nodes, they are able to suppress noise by identifying them as noise if the data are outside the radius or do not satisfy the minimum number of neighbors.

### 2.2. Appearance Feature Extraction

**Figure 4:**
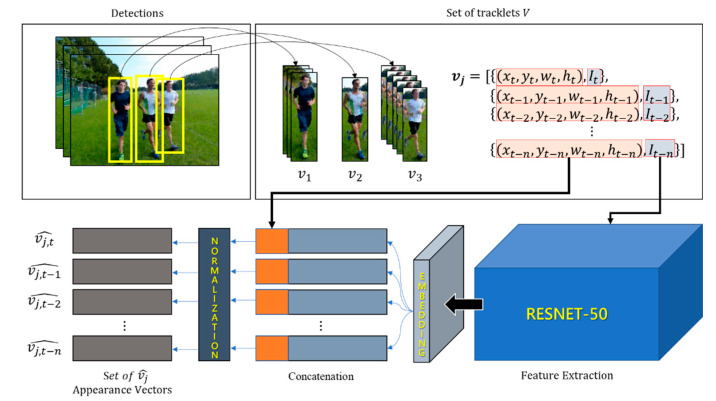
Preprocessing of raw frames for tracklet association by DBSCAN.

The set *V* of the detected objects is defined as the union set of tracklets consisting of object *j* detected in frames *t* to *t-n*. Here, *t* and *n* are defined as the current frame index and the *n*-th previous index, respectively, and *j* is the index assigned to the object. The object’s tracklet, *v_j_*, consists of the coordinates (*x*, *y*, *w*, *h*) of the bounding box of the detected object and its raw image frame. RESNET-50 [40] was used to extract the appearance feature vectors for an application as inputs for DBSCAN. The size of the feature map created by RESNET-50 was IW × IH × 2048, in which IW and IH are the width and height of the output vector in proportion to the input image size, respectively. Although the size of the input of DBSCAN should be fixed, the output of RESNET-50 is dependent on the size of the input image, and the extracted feature vectors have high-dimensional channels of 2048, which is not suitable for effective clustering of the tracklets because of its low computational efficiency. Therefore, to build an end-to-end learning system by extracting the feature map more efficiently and reducing the input dimension, we added the vector embedding layer proposed by the triplet network [41] to the output terminal of RESNET-50. Figure 4 shows the appearance feature vector extraction and preprocessing flow for use as the DBSCAN input.

The added neural network, a vector embedding layer, is a fully connected layer structure that is designed to reduce the output dimension to ${\mathbb{R}}^{256\times 1}$, considering the computational efficiency of DBSCAN. Here, RESNET-50 uses previously learned weights, and the embedding layer performs fine-tuning based on the same triple loss used in the triplet network. In the track-clustering process, the extracted feature vector is concatenated with the bounding box coordinates of the object to include the location information of the object and then processed as normalization to generate $\hat{v_{j}}\in {\mathbb{R}}^{260\times 1}$ for DBSCAN input. Thereafter, for clustering using $\hat{V}$, which is a set of $\hat{v_{j}}$ vectors, the input is assumed to be a spatial coordinate system and used in the data association step.

### 2.3. Tracklets Association Using DBSCAN

**Figure 5:**
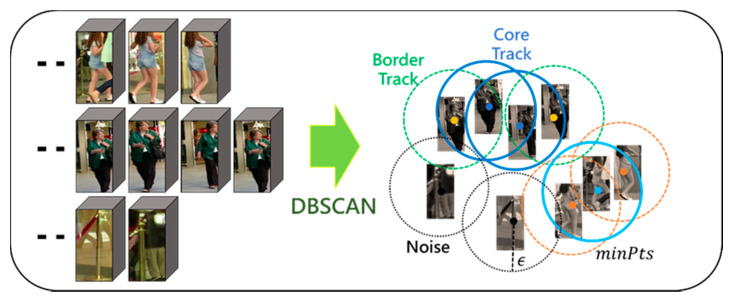
Illustration of object tracklets association process using DBSCAN.

The proposed DTAA is a method of clustering object tracklets based on DBSCAN, as discussed above. After obtaining the input vector of $\hat{v_{j}}\in {\mathbb{R}}^{260\times 1}$ for DBSCAN, data association is performed by clustering using DBSCAN. The object tracklet association process using DBSCAN for the proposed DTAA is shown in Figure 5. 

DTAA, which is transformed into a graph-based clustering method for MOT, uses $\epsilon^{*}\left(t\right)$. Temporal evolution is added to $\epsilon^{*}\left(t\right)$, as shown in (1), by considering the change over time to the minimum neighboring radius ϵ, a parameter of the existing DBSCAN, in the clustering process.

(1) $$\epsilon^{*}\left(t\right)=\mu e^{\frac{-{\left(t-1\right)}^{2}}{l^{2}}}$$

Here, *l* is the number of tracks constituting the object set *V*, and *µ* is the radius adjustment parameter. Objects detected long ago can be given low similarity by using $\epsilon^{*}\left(t\right)$ and can be transformed into the form of a Gaussian distribution. A track that satisfies the minimum neighboring *minPts* or higher based on $\epsilon^{*}\left(t\right)$ is defined as a core track, and a track that is clustered with the minimum neighboring but fails to expand below the standard is defined as a border track. The proposed tracklet association process is described in detail in Algorithm 1.

     **Algorithm 1.** Strengthening tracklet association process through DBSCAN clustering

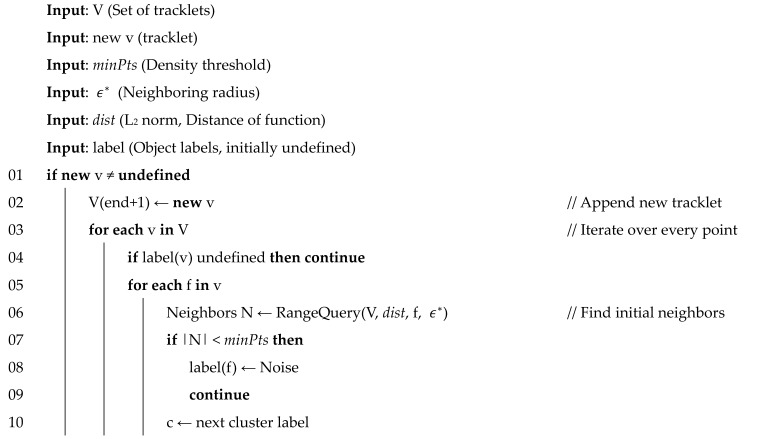


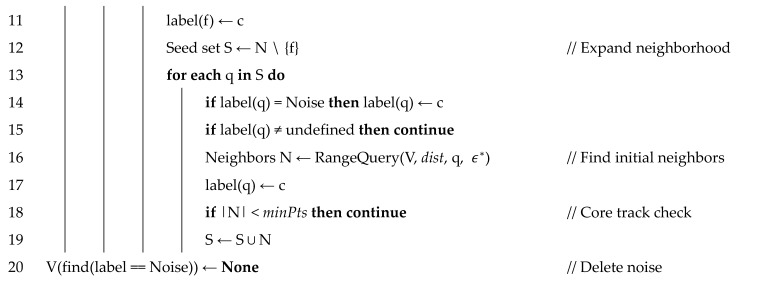



**Figure 6:**
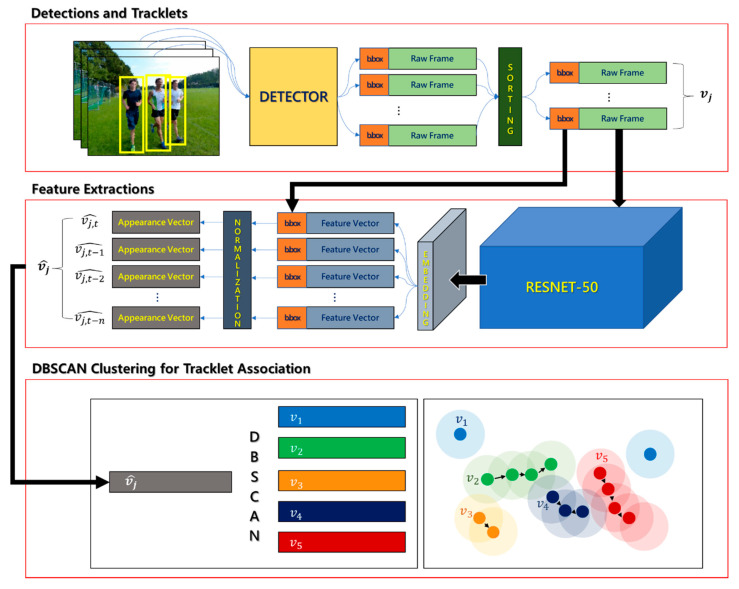
DBSCAN overall flow of multi-object tracking by the proposed DTAA.

The algorithm attempts to cluster when a new object or tracklet *v_j_* of an object *j* is applied as an input. In sequence, the algorithm calculates the distance to each object through the same RangeQuery function as in the conventional DBSCAN, and these objects are formed into a cluster when the number of neighboring objects is *minPts* or more within the radius $\epsilon^{*}$. After that, the objects not included in the cluster are regarded as noise and removed from set *V*. Figure 6 shows the flow of tracking multiple objects through the DTAA from object detection to clustering.

## 3. Experimental Results

The MOTChallenge 2015-16 (MOT15-16) benchmark dataset was used to evaluate the performance of the DTAA proposed in this study. Owing to the lack of more accurate object detection results using DNNs, the MOT15 benchmark is generally rated for low performance, even with state-of-the-art methods showing excellent performance in object tracking [42]. This dataset contains 11 training and test scenarios and evaluates the trajectory completeness for pedestrians. The training data included 500 object tracks and 39,905 bounding boxes. In contrast, the MOT16 dataset consists of 14 scenarios, 1342 tracks, and 292,733 bounding boxes. Standard MOT metrics were used for the evaluation. 

The MOT accuracy (MOTA), a representative MOT performance index, comprehensively evaluates the missed detection rate, false alarm rate (FAR), and IDS, as shown in (2).

(2) $$\mathrm{MOTA}=1-\frac{\sum_{t}\left(m_{t}+fp_{t}+mme_{t}\right)}{\sum_{t}g_{t}}$$

Here, $m_{t}$, $fp_{t}$, and $mme_{t}$ are three error sources, defined as the number of missing objects, number of false positives, and number of mismatch errors in the *t*-th frame. $g_{t}$ is the number of ground truths of the object to be detected. The MOTA metric is the most representative indicator for evaluating the MOT; however, as mentioned in [43], the following limitations exist. Because the threshold for object detection is very sensitive to the ground truth, it becomes too dependent on detections, and it is not easy to verify the overall robustness against noise such as Frag or IDS indices. Accordingly, we first compared the tracking performance based on MOTA and additionally conducted an evaluation focusing on the number of Frag, IDS, and FAR.

In addition, performance comparisons with conventional tracking methods were performed. To confirm the possibility of expanding the use of the proposed method, the DTAA was added to the end of the existing tracking algorithms in the form of late fusion as a postprocessor, and the verification task of its performance improvement was also conducted. In general, IOU Tracker and SORT, which can be easily used for object tracking, and tracking-by-detection techniques such as structural sparse tracking (SST) [44], joint detection and embedding (JDE) [45], and Adopting Tubes to Track Multi-Object in a One-Step Training Model (TubeTK) [46] based on DNNs were selected and compared with these prior works. IOU Tracker, SORT, and SST used the public detection results of the MOT15 benchmark dataset, whereas JDE and TubeTK used private detection results. Default values were used for the parameters and learning weights of the techniques used in the comparative evaluation. The learning for feature extraction through the proposed DTAA was conducted based on the MARKET-1501 dataset, and the following optimized values were derived through strategic learning for the parameters used in the data association: maximum track length of 20, *µ* = 0.75, *minPts* = 2. The comparative evaluation results of the proposed DTAA when used alone as a tracker in MOT and when incorporated with the existing trackers as a postprocessor are shown in Table 1 and Table 2, respectively.

**Table 1:** Tracking performance comparisons with MOT15 when the DTAA was used alone; best values are depicted in bold.

**Tracker**	**MOTA** **↑**	**FAR** **↓**	**IDS** **↓**	**Frag** **↓**	**Hz** **↑**
**Public**					
SORT [27]	26.0	1.20	779	1171	100+
IOU Tracker [28]	25.8	1.53	689	1120	100+
SST [44]	31.5	1.86	1262	1542	10
DTAA(ours)	26.8	**0.93**	**421**	879	30
**Private**					
JDE [45]	**35.5**	3.68	520	**823**	15
TubeTK [46]	59.6	1.02	858	1103	6

**Table 2:** Tracking performance comparisons when the DTAA was added to the state-of-the-art trackers as a postprocessor.

**Tracker**	**MOTA** **↑**	**FAR** **↓**	**IDS** **↓**	**Frag** **↓**
**MOT15** [36] **Public**				
SORT [27] + DTAA	27.5 (+5.7%)	1.00 (−16.7%)	442 (−48.4%)	879 (−4.9%)
IOU Tracker [28] + DTAA	26.4 (+2.3%)	1.42 (−7.2%)	518 (−24.8%)	751 (−2.9%)
SST [44]+ DTAA	33.7 (+7.0%)	1.39 (−25.3%)	717 (−3.2%)	1112 (−7.9%)
**Private**				
JDE [45] + DTAA	39.1 (+10.1%)	3.31 (−10.1%)	374 (−28.1%)	685 (−6.8%)
TubeTK [46] + DTAA	58.4 (−0.2%)	0.98 (−4.7%)	798 (−32.2%)	851 (−7.8%)
**MOT16** [37] **Public**				
SORT [27] + DTAA	22.6 (−)	2.15 (−60%)	1366 (−65%)	4713 (−53%)
IOU Tracker [28] + DTAA	27.5 (+1.3%)	0.20 (−12%)	751 (−21%)	841 (−13%)
SST [44] + DTAA	27.9 (+3.4%)	1.30 (−14%)	1095 (−14%)	2786 (−6.2%)
**Private**				
JDE [45] + DTAA	72.8 (−0.9%)	1.05 (−16%)	1248 (−6.3%)	1510 (−32.1%)
TubeTK [46] + DTAA	73.5 (+0.1%)	1.02 (−12.1%)	653 (−12.2%)	1123 (−8.4%)

When tracking objects using the DTAA alone, the highest level of MOTA could not be achieved, but FAR, IDS, and Frag were significantly lowered, which was our objective. In particular, the level of MOTA of the proposed tracker was similar or slightly higher than that of SORT and the IOU Tracker, whereas the indicators of FAR, IDS, and Frag showed significantly lower values, confirming that the tracking performance was considerably improved. Moreover, when DTAA was used as a postprocessor, the MOTA index improved by 2–10%, FAR decreased by up to 25% or more, and IDS and Frag decreased by almost 50%. In addition to improving the performance of existing tracking algorithms through the filtering effect, the FPS reduction rate as the amount of calculation increases was approximately 10% to 20%, confirming that the calculation speed did not significantly decrease owing to the additional process of DTAA.

**Figure 7:**
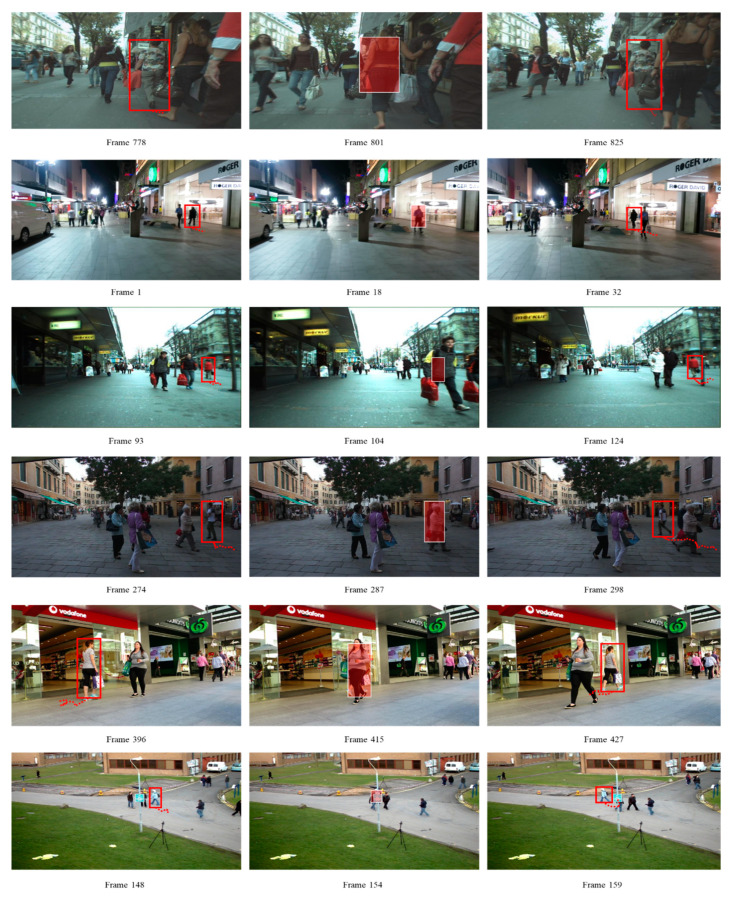
Exemplary tracking results using SST + DTAA (ours).

The result of expanding the proposed DTAA shown in Table 2 is remarkable. It is possible to perform more complete object tracking by removing noise with the DTAA from the trajectory of the object derived from the existing system without placing a large load on the data throughput. In all of the latest techniques, SORT and IOU Tracker, FAR, IDS, and Frag significantly decreased, whereas MOTA slightly increased. Even in the MOT techniques based on the private detection result, which have excellent detection performance, the tracking performance was similarly improved in terms of MOTA when the proposed DTAA was added as a postprocessor. Therefore, it was verified that even in the latest techniques, DTAA can be an augmentation system that can increase the robustness of the MOT algorithms and maximize the performance. Figure 7 shows some of the test evaluation results of the SST technique supplemented with DTAA.

An additional evaluation was conducted to verify the robustness against noise based on the processed data created by arbitrarily inserting noise into the previously used MOT15. To simulate the environment for temporal occlusion, data representing missed detection were created by randomly removing the detection results, and data representing noisy detection were created by adding noise to the bounding box of the detected object. In the data representing missed detection, the bounding box of the detected object was deleted with the probability of α%, and in the data representing the noisy detection, β% pixels were added to the bounding box of the detected object according to the intensity. Table 3 and Table 4 show the results of the robustness evaluation of SORT, IOU Tracker, and SST and their results when the proposed DTAA was added. As a result of all evaluations in a noisy environment, it was possible to maintain higher robustness, and the MOTA was increased by 5–10% on average compared to the existing method, regardless of the noise intensity or method, and IDS and Frag were reduced by approximately 30–40%. Although the overall performance was low owing to the effect of noise, practical applications are expected to achieve a large performance improvement.

**Table 3:** Comparisons of tracking performance when the DTAA was added to the state-of-the-art methods under noisy conditions considering missed detection with α = 10%.

**Tracker**	**MOTA** **↑**	**FAR** **↓**	**IDS** **↓**	**Frag** **↓**
SORT [27]	18.6	0.90	859	1906
IOU Tracker [28]	19.9	1.17	1306	1722
SST [44]	23.2	1.38	1240	1682
SORT [27] + DTAA	18.6 (−)	0.90 (−)	707 (−17.7%)	1477 (−22.5%)
IOU Tracker [28] + DTAA	22.3 (+12.1%)	1.01 (−13.7%)	503 (−61.5%)	1566 (−9.1%)
SST [44]+ DTAA	24.9 (+7.3%)	1.10 (−20.3%)	761 (−38.6%)	1432 (−14.9%)

**Table 4:** Comparisons of tracking performance when the DTAA was added to the state-of-the-art methods under noisy conditions considering noisy detection with β = ±15%.

**Tracker**	**MOTA** **↑**	**FAR** **↓**	**IDS** **↓**	**Frag** **↓**
SORT [27]	12.8	0.70	954	1454
IOU Tracker [28]	7.5	1.41	1192	2340
SST [44]	13.8	0.84	1046	1762
SORT [27] + DTAA	14.4 (+12.5%)	0.40 (−42.8%)	399 (−58.2%)	788 (−45.8%)
IOU Tracker [28]+ DTAA	10.8 (+44.0%)	1.12 (−20.6%)	676 (−43.3%)	1977 (−15.5%)
SST [44] + DTAA	15.1 (+9.4%)	0.76 (−9.5%)	785 (−24.9%)	1482 (−15.9%)

## 4. Conclusions

Recently, MOT techniques have attracted attention owing to the breakthrough development of autonomous driving and smart video surveillance systems. The main approach developed for tracking is a paradigm that identifies objects based on DNNs and tracks objects by analyzing the target identity in a video. Although significant technological advances have been made, it is still difficult to track objects in an environment in which occlusion is present. In this paper, we propose a strategy to enhance the performance of existing tracking techniques while solving the problem of noise vulnerability and operational efficiency degradation occurring in MOT. The proposed DTAA integrates the object feature vector and bounding box coordinates extracted through the CNN into a low-dimensional vector, mimics the graph model at the data association step, and clusters it with DBSCAN to estimate the trajectory of the object. Through test evaluation, when the proposed technique was used as a tracker alone, its performance indices of IDS and Frag were improved when compared with the existing tracking techniques. In particular, when additionally incorporated with conventional trackers as a postprocessor, significant performance improvement and noise suppression were observed. Thus, applications in industrial environments, such as actual pedestrian analysis and surveillance security systems, are expected.

However, the computational efficiency decreases as the number of objects to be tracked increases, and the high IDS between similar objects remains a problem to be solved. In order to make the proposed tracker more stable and reliable, we plan to increase the learning efficiency of CNN through the development of the scheme that effectively reduces the dimension of the embedding vector, and continue research on how to incorporate graph-based data association techniques into the clustering process.

## Data Availability

The data presented in this study are available on request from the corresponding author.
